# Transport properties of carboxylated nitrile butadiene rubber (XNBR)-nanoclay composites; a promising material for protective gloves in occupational exposures

**DOI:** 10.1186/2052-336X-12-51

**Published:** 2014-02-28

**Authors:** Mostafa Mirzaei Aliabadi, Ghasem Naderi, Seyed Jamaleddin Shahtaheri, Abbas Rahimi Forushani, Iraj Mohammadfam, Mehdi Jahangiri

**Affiliations:** 1 Department of Occupational Health Engineering, School of Public Health, Tehran University of Medical Sciences, Tehran, Iran; 2 Iran Polymer and Petrochemical Institute, P.O. Box: 14965/115, Tehran, Iran; 3 Department of Occupational Health Engineering, School of Public Health, Institute for Environmental Research, Tehran University of Medical Sciences, Tehran 14155-6446, Iran; 4 Department of Biostatistics, School of Public Health, Tehran University of Medical Sciences, Tehran, Iran; 5 Department of Occupational Health Engineering, Faculty of Health, Hamadan University of Medical Sciences, Hamadan, Iran; 6 Department of Occupational Health Engineering, School of Public Health and Nutrition, Shiraz University of Medical Sciences, Shiraz, Iran

**Keywords:** Nanocomposite, Chemical protective barrier, Swelling, Solvent transport properties

## Abstract

This study was conducted in response to one of the research needs of National Institute for Occupational Safety and Health (NIOSH), i.e. the application of nanomaterials and nanotechnology in the field of occupational safety and health. In order to fill this important knowledge gap, the equilibrium solubility and diffusion of carbon tetrachloride and ethyl acetate through carboxylated nitrile butadiene rubber (XNBR)-clay nanocomposite, as a promising new material for chemical protective gloves (or barrier against the transport of organic solvent contaminant), were examined by swelling procedure. Near Fickian diffusion was observed for XNBR based nanocomposites containing different amounts of nanoclay. Decontamination potential is a key factor in development of a new material for reusable chemical protective gloves applications, specifically for routine or highly toxic exposures. A thermal decontamination regime for nanocomposite was developed for the first time. Then, successive cycles of exposure/decontamination for nanocomposite were performed to the maximum 10 cycles for the first time. This result confirms that the two selected solvents cannot deteriorate the rubber-nanoclay interaction and, therefore, such gloves can be reusable after decontamination.

## Introduction

Chemical protective gloves are extensively used as solvent barriers to prevent or lower the exposure to hazardous chemicals [1]. In such applications, permeability of gloves against solvents is of great practical concern. This process is commonly described by a three-step mechanism: (i) dissolving of the penetrant molecules in the polymer, (ii) diffusion of the penetrant molecules through the polymer; and finally (iii) desorption of the penetrant molecules on the inner surface of the gloves [2-5]. There are many factors influencing the intensity of solvent permeability including; the affinity of polymer molecule to penetrant molecule, size, shape, and the nature of the penetrant molecules [6,7], presence of fillers, and solvent-induced structural changes duo to the successive cycles of exposure/decontamination [8].

Nowadays, polymer-clay nanocomposites are widely used due to their outstanding properties such as chemical resistance, thermal stability, mechanical strength, and inflammability [9,10]. The enhancement of solvent barrierity in these nanocomposites is attributed to the implanted impermeable platelet structure of nanoclay, which forces the penetrant molecules to wiggle around them and thus creating a tortuous route for passing of penetrant [11-13]. Therefore, wherever good barrierity is needed such as chemical protective gloves, commercial applications of these material products seem to be promising. In addition, this study is a response to one of the research needs of National Institute for Occupational Safety and Health (NIOSH), i.e. the application of nanomaterials and nanotechnology in the field of occupational safety and health [14-17].

The present paper focuses on the transport properties of a nanocomposite based on carboxylated nitrile butadiene rubber (XNBR)-nanoclay. Liquid immersion measurements were conducted for different nanocomposites using two neat organic solvents (i.e. carbon tetrachloride (CCl_4_) and ethyl acetate). Diffusion and solubility coefficients of these solvents were estimated in the nanocomposite samples. In addition, a thermal decontamination regime for the nanocomposite was developed for the first time. Then, the successive cycles of exposure/decontamination of nanocomposite were conducted to a maximum 10 cycles for the first time. The results were used to predict the extent of rubber–nanoclay interaction after increasing the number of use/decontamination of gloves in the presence of nanoclay.

### Experimental

XNBR latex used in this research was SYNTHOMER 6617 which was kindly supplied by SYNTHOMER company (SYNTHOMER, Malaysia). Ingredients of vulcanizations and selected solvents (Carbon tetrachloride [solubility parameter, 17.6 MPa^1/2^] and Ethyl acetate [solubility parameter, 18.6 MPa^1/2^]) were provided by the standard suppliers. Listed purities were at least 99 percent for the Carbon tetrachloride and Ethyl acetate. Pristine sodium montmorillonite (Na^+^ MMT), with a cationic exchange capacity (CEC) of 92.6 mequiv/100 g, was obtained from Southern Clay Products, Texas, USA.

### Nanocomposite preparation

Based on the protocol designed for preparation of the nanocomposite, firstly, a 5% aqueous suspension of clay was prepared by a mechanical stirrer. The pH of this dispersion was adjusted to the pH of XNBR latex by adding KOH (potassium hydroxide) solution. In order to counteract the sensitizing effect of layered silicates, sufficient amounts of Emulvin WA was added to the aqueous dispersion of clay and then, it was vigorously stirred for a given time. Subsequently, the as-prepared clay aqueous dispersion was added into the XNBR latex, and the mixture was stirred using a magnetic stirrer at room temperature for a period of 24 hours. The procedure was similarly repeated to prepare samples containing different amounts of clay; i.e. 3, 6, 9 phr (parts per hundred of rubber). Thereafter, curative ingredients, which were prepared in the colloidal form, were added to the XNBR nanocomposite and the mixture was further stirred for 24 hours. A glove former was then used to produce films of nanocomposites by coagulating dipping. In practice, glove former was dipped in a 40% calcium nitrate solution (as coagulant) for 15 seconds and dried at 100°C for 3 minutes. It was then cooled until temperature reached below 65°C. This coagulant coated former was dipped into the latex nanocomposite and was dwelled for one minute. The sample was kept at room temperature for 3 minutes to form a gel and it was then leached with the water at 45°C. Finally, the dipped samples were cured in the hot air oven at 130°C.

In the following sections for simplicity, the samples are denoted as XNBR_MMT_, where the subscript MMT indicates the amount of MMT used (i.e. 0, 3, 6, and 9 phr). Accordingly, XNBR_6_ represents XNBR nanocomposite containing a nanoclay loading of 6 phr. The formulation of compounds is given in Table 1.

**Table 1:** Designation and formulation details of XNBR-clay nanocomposite

**Designation**	**XNBR rubber (dry parts/phr)**	**Sulfur (phr)**	**ZDEC**^**a **^**(phr)**	**Zinc oxide (phr)**	**5% clay dispersion**
XNBR_0_	100	1	1	3	0
XNBR_3_	100	1	1	3	3
XNBR_6_	100	1	1	3	6
XNBR_9_	100	1	1	3	9

^a^Zinc diethyldithiocarbamate.

### Swelling procedure

In each experiment the circular samples of 2 cm diameter of were punched out using a sharp edged steel die. The average thickness of nanocoposite samples was 0.35 ± 0.02 mm and difference in samples thicknesses was not significant. In order to mimic the worst-case conditions for protective gloves during usage, the punched samples were weighted and placed in a screw tight glass bottle containing liquid. At specified time intervals, samples were picked up from the test bottle and blotted to remove excess solvent, and then were weighed. The weighting procedure was carried out fast enough within 30-40 seconds to minimize the errors raised from the solvent evaporation. The samples were placed back into the test bottle after each weighting. The results of swelling measurements were expressed as the mass of solvent uptake. The reported values for mass uptake are the average of three swelling experiments. It has been practically observed that the temperature at the inside of a glove can approach to the body temperature (37°C), therefore, the aforementioned procedure was performed at 37°C [18-20].

### Evaluation of decontamination efficiency

Swelling procedure was used to contaminate (exposure) XNBR-clay nanocomposites by the selected solvent. Once the required time for maximum mass uptake was elapsed, which was determined previously by swelling procedure, the sample was removed from the test bottle and was placed into a hot-air oven for decontamination at 100°C for 16 hours. Decontamination conditions were selected based on the work of Vahdat and Delaney [21], which were the same as Gao study [22].

After the completion of decontamination, the effectiveness of the procedure was calculated simply by the change in weight percentage:

(1) $$\%\;\mathrm{Decontamination}\;\mathrm{efficiency}\;=\;\frac{\mathrm{Weight}\;\mathrm{loss}}{\mathrm{Weight}\;\mathrm{gain}}\times 100$$

In the equation (1), the weight gain is the difference of weight before and after of the decontamination process. Similarly, the weight lost was considered as the difference between the weight of the contaminated sample prior- and post–decontamination.

### Examination of polymer–nanoclay interactions

A unique approach based on the repeated cycles of contamination and decontamination was used to examine the extent of the polymer–nanoclay interactions. To this end, an ascending number of exposure/decontamination cycles up to maximum 10 cycles was imposed on the samples. Hence, the change in permeability coefficient of the nanocomposite film was demonstrated. Subsequently, it was determined whether these gloves would be reusable. To do this, swelling procedure was conducted until maximum mass solvent uptake accomplished. Then, sample was removed from the test bottle and the liquid on the surface of the samples was rubbed off and the decontamination procedure was started.

## Results and discussion

### Swelling behavior

The dynamic swelling characteristics of a nanocomposite film includes; the solvent sorption rate, the transport mechanism that regulates solvent sorption, the rate of reaching to the equilibrium swelling, and the sorption of solvent or equilibrium solubility.

**Figure 1:**
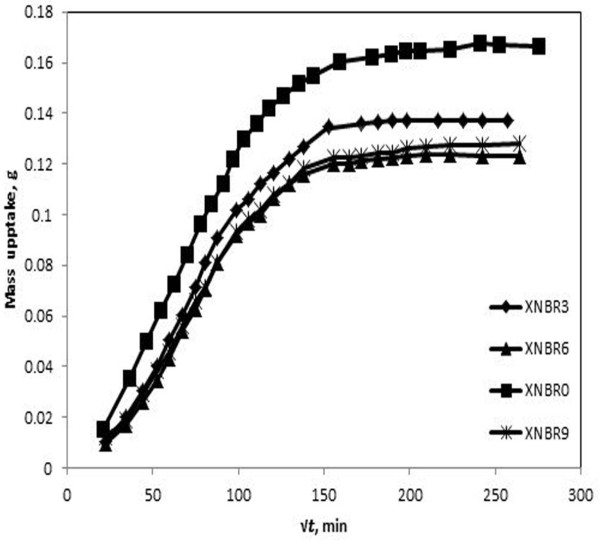
**Sorption curves of CCl**_**4 **_**with nanocomposites at different nanoclay loading.**

The swelling curves expressed as the mass uptake of solvent versus square root of time,√*t*, at 37°C for the nanocomposites– CCl_4_ are given in Figure 1. The sorption curves of nanocomposites-ethyl acetate pair not shown here. After attainment of the equilibrium swelling, experiments were continued for longer times to ensure complete equilibrium. It was observed that the swelling proceeds with a high rate of solvent uptake, since the concentration gradient of the permeant in material is large. Then, the rate of solvent uptake decreases, owing to decreasing trend in concentration gradient of the permeant molecules. To examine the mechanism of solvent transport, some attempts were made to fit the swelling results in heuristic expression [23,24]:

(2) $$\log\left(\frac{M_{t}}{M_{\infty }}\right)=\log\;k\;+\;\mathrm{nlog}\;t$$

Where M_t_ and M_∞_ are the values of mass uptake at time t and at equilibrium t_∞_, respectively. The value of k is constant characteristics of the polymer-solvent system and illustrates the interaction between polymer and solvent. The values of k and *n* were found by linear regression analysis are summarized in Table 2. The values of n determine the type of the transport mechanism based on the relative mobility rate of permeant and the polymeric strands. For instance, when n = 0.5 the process is called Fickian (or Case I). Here, the rate of permeant mass uptake is proportional to $\sqrt{t}$ and the concentration gradient of the permeant molecules is the main factor that controls the diffusion. If the rate of permeant mass uptake is directly proportional to time, the process is referred to non-Fickian (or Case II) and hence n equals to1. However, the intermediate values i.e. 0.5 < n <1 are the characteristic of anomalous transport mechanism, which the rate of diffusion is equal to the rate of the relaxation of polymeric strands [25].

**Table 2:** **The value of *n *** **and *k *** **at different nanoclay loading in selected solvents**

	**Carbon tetrachloride**		**Ethyl acetate**	
**Sample**	***n*** **± 0.01**	***k*** **± 0.007**	***n*** **± 0.01**	***k*** **± 0.007**
XNBR_0_	0.510	0.196	0.507	0.590
XNBR_3_	0.555	0.158	0.590	0.333
XNBR_6_	0.582	0.110	0.614	0.322
XNBR_9_	0.554	0.152	0.537	0.642

As can be seen from Table 2, regarding to the value of n for neat polymer (XNBR_0_), the both solvents exhibit Fickian mode. Upon adding of filler the diffusion behavior slightly deviated from the Fickian trend. It observed that the value of n for all of the nanocomosites vary from 0.507 to 0.614, indicating that the related transport mode would be expected as the near Fickian sorption. The *k* value implies the interaction between nanocomosites-solvent system and as would be expected, its value decreased with increase of the filler loading. Ethyl acetate is a polar solvent and hence possibly due to the dipole-dipole interaction with the polar groups of rubber segments, the *k* values are higher for ethyl acetate compared to CCl_4_ in all experiments [26]. The solvent uptake rate represents the amount of solvent absorbed per unit of time into the nanocomposite. In the case of Fickian mechanism, the rate of reaching to equilibrium swelling can be determined by diffusion coefficient. In order to obtain the diffusion coefficient (D) of the nanocomposite-solvent system, the values of solvent uptake, before attaining 50% of equilibrium (i.e. $0\leq \frac{M_{t}}{M_{\infty }}\leq 0.5$), were fitted to the equation (3) [25,27,28]:

(3) MtM∞=4hDtπ12

Where *h* is the thickness of nanocomposite film.

The estimated values of diffusion coefficient *D* are presented in Table 3.

**Table 3:** Solubility [S], Diffusion [D], and Permeability [P] coefficient values of XNBR- nanocomposites

	**CCl**_**4**_			**Ethyl acetate**		
**Sample**	**S (*g/cm***^**3**^**)**	***D*** **× 10**^**7**^**(*cm***^**2**^**/ *s *)**	***P*** **× 10**^**7**^**( *g */*cm***^**2**^**- *s *)**	**S (*g/cm***^**3**^**)**	***D*** **× 10**^**7**^**(*cm***^**2**^**/ *s *)**	***P*** **× 10**^**7**^**( *g */*cm***^**2**^**- *s *)**
XNBR_0_	1.16	3.62	4.19	1.72	5.25	9.03
XNBR_3_	0.943	3.54	2.97	1.24	4.39	5.44
XNBR_6_	0.859	2.71	2.32	1.17	4.05	4.73
XNBR_9_	0.892	2.84	2.53	1.17	4.39	5.13

Equilibrium solubility *S* (g/cm^3^) of the selected solvent in the nanocomposite is obtained by equation (4):

(4) $$\begin{array}{l} \mathrm{Equilibrium}\;\mathrm{solubility}\left(g/cm^{3}\right)\\ \;=\frac{\mathrm{Mass}\;\mathrm{uptake}}{\mathrm{Volume}\;\mathrm{of}\;\mathrm{the}\;\mathrm{dry}\;\mathrm{sample}} \end{array}$$

It can be seen from the data in Table 3 that the values of S and *D* for ethyl acetate are higher than those of CCl_4_ in all nanocomposites.

The higher value of *S* could be attributed to this fact that the molecular weight of ethyl acetate is lower than CCl_4_ in each experiment.

The higher value of *D* could be related to the existence of difference in the solubility parameter (thermodynamic affinity) of the solvent and the polymer. The solubility parameter of CCl_4_, ethyl acetate, and the polymer are 17.6, 18.6, and 20.5 MPa^1/2^ respectively. The difference in the solubility parameter of CCl_4_ and polymer (2.9 MPa^1/2^) is lower than for ethyl acetate and polymer (1.9 MPa^1/2^). Therefore, the thermodynamic affinity of ethyl acetate is higher for XNBR polymer. Also, moderate hydrogen-bonding tendency of ethyl acetate along with the hydrophilic features of the surface of nanoclay increase the interaction between ethyl acetate and nanocomposite.

**Figure 2:**
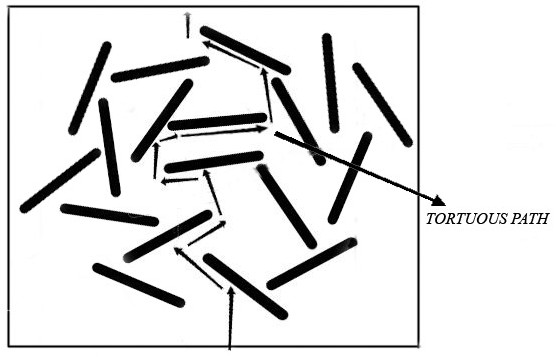
Schematic representation of how tortuous zigzag path can longer the traveled distance of solvent molecules through the nanocomposite.

From Table 3 it can be seen that the values of *S* and *D* decrease in the presence of nano filled samples compared to the neat ones. The decrease in S value may be attributed to the reduced volume fraction of permeable polymer in the presence of impenetrable silicate layered i.e. the reduction of available cross sectional area for diffusion. The latter can be explainable by the tortuous zigzag path (Figure 2) created by the clay nanolayers that enforce the solvent molecule to bypass the impenetrable silicate layered and thereby, increasing the traveled distance. In addition, it is widely accepted that transport properties of permeant molecules increase with the mobility of rubber chain. In nanocomposite, due to the nanoconfinement of very small rubber chain by comparable larger nanoclay, the effective surface area of nanoclay increase to create the strong rubber-nanoclay interactions. This will reduce the availability of free volume and restrict the mobility of the rubber chain segments even at the small amount of loading (<3 phr). However, the extent to which transport properties are affected by adding nanoclay is a function of the difference of solubility parameters (between neat polymer and permeant solvent) and the amount of filler. The significant decrease in the permeability coefficients of the selected solvent was observed at 3 phr nanoclay loading, due to the exquisite dispersion of nanoclay at rubber matrix. However, the XNBR_6_ showed the maximum reduction in permeability coefficient, because of the higher amount of nanoclay loading. Also, permeability coefficients were increased as a function of nanoclay loading. This may be ascribed to the aggregation of nanocaly at higher loading.

Assuming near-Fickian diffusion, the following equation (5) can be used to define the steady state permeability coefficient, *P* (g/cm-s) [13,29]:

(5) P=DS

Where the *D* and *S* have already been defined. The permeability results are given in Table 3. It is evident that the permeability coefficient value decreases as the filler loading in the composite is increased. The dispersed nanoclay hinders the mobility of the permeant molecules across the polymer strands. Accordingly, as the filler loading in the composite was increased a lower value of *D* was obtained. The value of *S* also illustrates the same direction. Since *P* is the net product of *D* and *S*, the *P* value exhibits a decrement as the filler loading in the composite increases.

**Figure 3:**
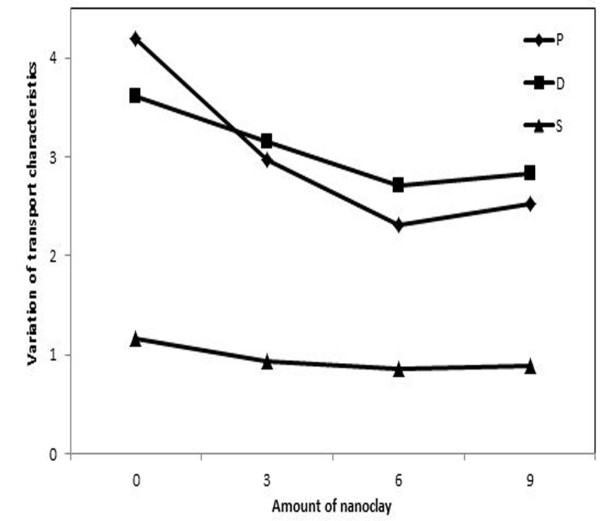
**Variation of transport characteristics of CCl**_**4 **_**against XNBR-nanocomposite at different nanoclay loading.**

As can be seen in Figure 3, the drop in permeability of CCl_4_ is mainly due to the drop in diffusivity. This implies that, in XNBR the dispersed nanoclay has a more effect on diffusion coefficient than the equilibrium solubility. This desirable change in barrier properties is owing to the waste of energy of the permeant molecules as they have to bypass the impenetrable silicate layered and thereby, the increase of the traveled course.

### Evaluation of decontamination efficiency

The feasibility for decontamination is an important issue in the development of a new material for reusable chemical protective gloves applications specifically for routine or highly toxic exposures. Therefore, materials are essentially expensive to be regarded as disposable.

Since XNBR_3_ containing the minimum amount of nanoclay shows the efficient decrease in permeability coefficient of ethyl acetate compared to the neat polymer, the XNBR_3_-ethyl acetate pair was selected for decontamination procedure. Also, for comparison with XNBR_3_, the same exposure/decontamination procedure was carried out on the neat polymer (XNBR_0_).

**Figure 4:**
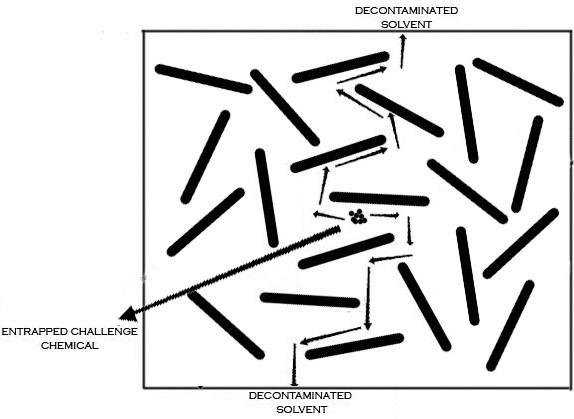
Schematic representation of tortuous path on the reverse diffusion of entrapped challenge chemical.

The results of thermal decontamination (for 16 hours at 100°C) of the samples based on the weight change are summarized in Table 4. It is believed that the difference between the run 0 and 1 is due to the extraction of rubber additives (curing agents) and low molecular weight chain of the rubber upon the first exposure of ethyl acetate to composite sample [27,28]. Hence, the numerator, i.e. weight loss, will be greater than the actual value and hence the decontamination efficiency will be too large. At this decontamination condition, decontamination efficiency of XNBR_3_ was lower than the neat polymer. This discrepancy is attributed to the effect of tortuous path on the reverse diffusion of entrapped challenge chemical (Figure 4). When the matrix of the glove is contaminated, the process will reverse because the concentration gradient is reversed. This can be affected by removing the surface contamination. Therefore, the reverse diffusion represents an important decontamination step if an enough time is given. Thus, it seems that the time requirement for the complete decontamination of any nanocomosite will be longer than the corresponding neat polymer. As the tortuous path can hinder the diffusion of permeant molecules in the contamination (exposure) process of XNBR_3_, the same effect delays the release of entrapped permeant molecules in the reverse diffusion of decontamination process and prolong the decontamination time in the same temperature. The results in Table 4, shows that the contamination cannot be removed completely in 16 hours from XNBR_3_ composite. With increase of the time up to 24 hours (Table 4) the decontamination was effective in removing roughly 99 percent of the ethyl acetate from XNBR_3_ composite.

**Table 4:** **Thermal decontamination of ethyl acetate from XNBR composite at 100**°C **for different time periods**

**Run**	**Decontamination efficiency%**		
	**16 hours**		**24 hours**
	**XNBR**_**0**_	**XNBR**_**3**_	**XNBR**_**3**_
0^a^	101.5	95.1	101.1
1^b^	99.3	94.2	99.8
2^c^	99.4	93.9	99.6

^a^The initial sample.

^b^The first reuse.

^c^The second reuse.

### Examination of polymer–nanoclay interactions

Because the XNBR_3_ containing the minimum amount of nanoclay showed the maximum decrease in permeability coefficient of ethyl acetate compared with neat polymer, the XNBR_3_-ethyl acetate pair was selected for the examination of polymer–nanoclay interaction.

**Figure 5:**
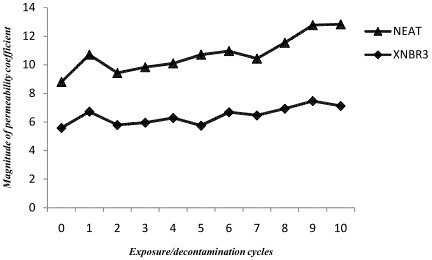
**Variations of permeability coefficients of ethyl acetate against XNBR**_**3 **_**after exposure/decontamination cycles.**

Change in the transport properties of XNBR_3_–ethyl acetate pair after exposure/decontamination cycles are provided in Table 5. In comparison with the new sample, after 10 exposure/decontamination cycles, the permeability coefficient of nanocoposites was increased on average 15 percent of their original value. As can be seen in Figure 5, this increase mainly occurs in the first and the ninth exposure/decontamination cycle. The significant increase in the permeability coefficients after the first cycle is due to the aforementioned factors, the extraction of rubber additives, *etc*. To determine whether the variation in permeability coefficients can occur in any XNBR-ethyl acetate systems, regardless of the presence of nanoclay, a similar exposure/decontamination procedure (up to 10 cycles) was also conducted for the neat polymer as reference. As it can be observed in Table 6, the ratio of both diffusion and permeability coefficients of the XNBR_3_ to the neat rubber approximately stay consistent in all exposure/decontamination cycles. This result confirms that solvent is not able to deteriorate the rubber-nanoclay interaction, promising that gloves could be reusable after decontamination.

**Table 5:** **Change in the transport properties (Solubility [S], Diffusion [D], and Permeability [P] coefficient values of) of XNBR**_**3**_**–ethyl acetate pair after exposure/decontamination cycles**

	**XNBR**_**3**_			**NEAT polymer**		
**Run**	**S (*g/cm***^**3**^**) *g/cm***^**3**^	***D*** **× 10**^**7**^**(*cm***^**2**^**/ *s *)**	***P*** **× 10**^**7**^**( *g */ *cm *- *s *)**	**(*g/cm***^**3**^**)**	***D*** **× 10**^**7**^**(*cm***^**2**^**/ *s *)**	***P*** **× 10**^**7**^**( *g */ *cm *- *s *)**
0	1.26	4.43	5.58	1.69	5.21	8.80
1	1.42	4.73	6.72	1.92	5.58	10.71
2	1.29	4.49	5.79	1.72	5.48	9.43
3	1.31	4.54	5.95	1.73	5.68	9.83
4	1.36	4.62	6.28	1.72	5.87	10.10
5	1.29	4.45	5.74	1.81	5.92	10.71
6	1.41	4.74	6.68	1.82	6.02	10.96
7	1.39	4.65	6.64	1.81	5.76	10.43
8	1.44	4.81	6.93	1.92	6.01	11.54
9	1.51	4.94	7.46	2.12	6.03	12.78
10	1.46	4.88	7.12	2.15	6.05	12.83

**Table 6:** **The ratio of both diffusion and permeability coefficients of the XNBR**_**3 **_**to the neat rubber after exposure/decontamination cycles**

**Run**	**0**	**1**	**2**	**3**	**4**	**5**	**6**	**7**	**8**	**9**	**10**
D_XNBR3_/D_neat_	0.85	0.85	0.82	0.80	0.79	0.75	0.79	0.81	0.80	0.82	0.81
P_XNBR3_/P_neat_	0.63	0.63	0.61	0.60	0.62	0.54	0.61	0.62	0.60	0.58	0.55

## Conclusion

In this study, the equilibrium solubility and diffusion of selected solvents in carboxylated nitrile butadiene rubber (XNBR)-clay nanocomposite, as a promising new material for chemical protective gloves or barrier against the transport of organic solvent contaminant, were examined by swelling procedure. By virtue of the favoring affinity between the rubber and the nanoclay, nanocomposites eventually lower the permeability coefficients of solvents. This occurs due to some events such as the creation of a zigzag path, the reduction in the availability of free volume, and the restriction on the mobility of rubber chain segments. Ethyl acetate permeability coefficients decreased significantly at 3 phr loading of nanoclay (5.44 × 10^- 7^g/cm ‒ s) in comparison with the neat polymer (9.03 × 10^‒ 7^g/cm ‒ s). For the same reason that permeability decreased due to the dispersed nanoclay, a longer time is required for the effective decontamination of absorbed chemicals from the nanocomposite compared to conventional nitrile gloves. Successive cycles of exposure/decontamination confirm that solvent are not able to demolish the rubber-nanoclay interaction, promising gloves could be reusable after decontamination.

## Competing interests

The authors declare that they have no competing interests.

## Authors’ contributions

The overall implementation of this study including design, experiments and data analysis, and manuscript preparation were the efforts of the first three authors. The fourth author helped in the experimental design and data analysis. Other authors participated in the synthesize and characterization of nanocompisites. All authors have read and approved the final manuscript.
